# A comparative analysis of metacommunity types in the freshwater realm

**DOI:** 10.1002/ece3.1460

**Published:** 2015-03-11

**Authors:** Jani Heino, Janne Soininen, Janne Alahuhta, Jyrki Lappalainen, Risto Virtanen

**Affiliations:** 1 Biodiversity, Finnish Environment Institute, Natural Environment CentreP.O. Box 413, FI-90014, Oulu, Finland; 2 Department of Geosciences and Geography, University of HelsinkiP.O. Box 64, FI-00014, Helsinki, Finland; 3 Department of Geography, University of OuluP.O. Box 3000, FI-90014, Oulu, Finland; 4 Finnish Environment Institute, Freshwater Centre, State of Surface WatersP.O. Box 413, FI-90014, Oulu, Finland; 5 Department of Environmental Sciences, University of HelsinkiP.O. Box 65, FI-00014, Helsinki, Finland; 6 Department of Biology, University of Oulu, Botanical MuseumP.O. Box 3000, FI-90014, Oulu, Finland

**Keywords:** Algae, invertebrates, lakes, macrophytes, metacommunity, multigroup analysis, streams

## Abstract

Most metacommunity studies have taken a direct mechanistic approach, aiming to model the effects of local and regional processes on local communities within a metacommunity. An alternative approach is to focus on emergent patterns at the metacommunity level through applying the elements of metacommunity structure (EMS; *Oikos*, 97, 2002, 237) analysis. The EMS approach has very rarely been applied in the context of a comparative analysis of metacommunity types of main microbial, plant, and animal groups. Furthermore, to our knowledge, no study has associated metacommunity types with their potential ecological correlates in the freshwater realm. We assembled data for 45 freshwater metacommunities, incorporating biologically highly disparate organismal groups (i.e., bacteria, algae, macrophytes, invertebrates, and fish). We first examined ecological correlates (e.g., matrix properties, beta diversity, and average characteristics of a metacommunity, including body size, trophic group, ecosystem type, life form, and dispersal mode) of the three elements of metacommunity structure (i.e., coherence, turnover, and boundary clumping). Second, based on those three elements, we determined which metacommunity types prevailed in freshwater systems and which ecological correlates best discriminated among the observed metacommunity types. We found that the three elements of metacommunity structure were not strongly related to the ecological correlates, except that turnover was positively related to beta diversity. We observed six metacommunity types. The most common were Clementsian and quasi-nested metacommunity types, whereas Random, quasi-Clementsian, Gleasonian, and quasi-Gleasonian types were less common. These six metacommunity types were best discriminated by beta diversity and the first axis of metacommunity ecological traits, ranging from metacommunities of producer organisms occurring in streams to those of large predatory organisms occurring in lakes. Our results showed that focusing on the emergent properties of multiple metacommunities provides information additional to that obtained in studies examining variation in local community structure within a metacommunity.

## Introduction

Community ecologists have recently shifted their focus from studying single local communities to considering a set of local communities in a region (Leibold et al. 2004; Logue et al. 2011). The main focus in studying such a metacommunity is still the structure of local communities, but the mechanisms are not assumed to be solely local, like biotic interactions, but that regional factors, like dispersal among sites, also affect local community structure (Leibold et al. 2004; Heino et al. 2015a). Hence, most of current community ecology research tries to disentangle the relative roles of local and regional processes on local community structure within a single metacommunity (Cottenie 2005; Meynard et al. 2013). An alternative means is to focus on the patterns emerging at the level of a metacommunity (Leibold and Mikkelson 2002; Presley et al. 2010; Newton et al. 2012; Dallas 2014).

Metacommunities show multiple patterns in space and time, ranging from those assuming underlying species interactions to those suggesting independent responses of species to environmental gradients (McIntosh 1995; Mittelbach 2012). These ideas were at the heart of ecology already in the first half on the 20th century, when vegetation ecologists disputed about the discrete versus continuous nature of variation in community structure along environmental gradients (for definitions of main concepts, see Table1). Clements (1936) argued for discrete community types (i.e., Clementsian gradients), whereas Gleason (1926) promoted the idea that single species respond independently to environmental gradients (i.e., Gleasonian gradients). This dispute never reached a final agreement (McIntosh 1995), although some plant and animal ecologists have thereafter favored more individualistic than discrete concepts of community variation (Austin and Smith 1989; Ricklefs 2008).

**Table 1:** A glossary of the main concepts dealt with in this article. See Leibold and Mikkelson (2002) and Presley et al. (2010) for additional information and methods for delineating metacommunity types

Concept	Definition
Boundary clumping	A measure that takes into account how the edges of species range boundaries are distributed along a dimension or an ordination axis (Leibold and Mikkelson 2002)
Checkerboards	A checkerboard pattern exists if species pairs have mutually exclusive distributions across a set of sites and such pairs occur independently of other pairs of species (Diamond 1975)
Clementsian	A gradient model where species respond to ecological gradients as groups, resulting in discrete communities (Clements 1936). Clementsian metacommunity type is one of the six main metacommunity types in our study
Coherence	A measure of the degree to which a pattern can be collapsed into a single dimension or an ordination axis (Leibold and Mikkelson 2002)
Evenly spaced	There are no discrete communities, but species ranges are arranged more evenly than what could be expected by chance (Tilman 1982)
Gleasonian	Species respond individualistically to underlying ecological gradients (Gleason 1926). Gleasonian metacommunity type is one of the metacommunity types in our study
Turnover	A measure of turnover in species composition along a dimension or an ordination axis. In the EMS framework, it measures the number of species replacements (Leibold and Mikkelson 2002)
Metacommunity structure	A combination of inferences from the significance of *coherence*, *turnover,* and *boundary clumping* (Leibold and Mikkelson 2002). In our study, we consider metacommunity structures synonymous to *metacommunity types*
Metacommunity type	See above. A metacommunity type can be defined as a pattern in a site-by-species matrix that is statistically significant from random expectations
Nestedness	A pattern where sites poor in species contain proper subsets of species from progressively richer communities (Patterson & Atmar 1986). In our study, nested metacommunity is one metacommunity type
Quasi-structure	Quasi-structures are intermediate metacommunity types. Quasi-nested metacommunity is the name for cases of significant positive coherence and nonsignificant turnover. Quasi-evenly spaced, quasi-Gleasonian, and quasi-Clementsian are the names for cases with positive coherence and positive turnover, and they can be distinguished based on boundary clumping (Presley et al. 2010)
Random	There are no clear gradients or discernible patterns in species distributions across a set of sites (Leibold and Mikkelson 2002)

Until recently emergent metacommunity patterns have been difficult to test owing to lack of suitable statistical methods. One modern approach is to test the fit of empirical data with multiple metacommunity structures simultaneously (Leibold and Mikkelson 2002; Presley et al. 2010). Those patterns can be illustrated by the three *elements of metacommunity structure* (i.e., coherence, turnover, and boundary clumping) and, subsequently, that information can be used to delineate *metacommunity types* (Leibold and Mikkelson 2002). The main metacommunity types are checkerboard (Diamond 1975), nested (Wright et al. 1998), evenly spaced (Tilman 1982), Gleasonian (Gleason 1926), Clementsian (Clements 1936), and random (Leibold and Mikkelson 2002). These metacommunity types are broad idealizations of nature, and hence, a number of subtypes can also be distinguished. Presley et al. (2010) suggested that the cases of significant positive coherence followed by nonsignificant turnover along an ordination axis can be considered as quasi-structures, with nonsignificant negative turnover referring to quasi-nestedness, and nonsignificant positive turnover to quasi-evenly spaced, quasi-Gleasonian, or quasi-Clementsian metacommunity structures that can be distinguished based on boundary clumping (Leibold and Mikkelson 2002).

Many, if not most, metacommunity patterns have typically been studied in isolation. While studies focusing on a single metacommunity pattern continue to provide important information about ecological communities, they may also fall short because they do not yield a comparative understanding of metacommunities (Presley et al. 2009; Meynard et al. 2013; Dallas 2014; Dallas and Presley 2014). Therefore, a simultaneous comparison of multiple patterns is necessary so that we can find “the best fit” patterns of metacommunity structure in various ecological systems (Leibold and Mikkelson 2002; Presley et al. 2010). There is thus an impetus to examine multiple metacommunity patterns simultaneously and reveal if observed metacommunity types are molded predictably by a set of ecological variables or if those metacommunity types are only products of context dependency (Lawton 1999). Context dependency may be caused by variations in regional species pools and underlying environmental conditions even for the same ecosystem type (Heino et al. 2012), it may lead to patterns that are temporally variable due to varying environmental conditions (Erős et al. 2014; Fernandes et al. 2014), and it may eventually result in situations where findings of metacommunity patterns are not easily transferable beyond the studied system (Heino et al. 2012). Such unpredictability results when we cannot detect any general relationships between metacommunity types and their underlying ecological characteristics, such as ecosystem type, trophic position of organisms or latitude. Hence, if metacommunity-level phenomena are as weakly predictable as many local community-level patterns (Lawton 1999), context dependency may hinder our attempts to generalize findings from one system to another (Heino et al. 2012).

Freshwaters provide suitable model systems for addressing questions related to the organization of metacommunities. First, those systems are embedded in the terrestrial matrix that is unsuitable for the development of the aquatic stages of freshwater organisms and, hence, delineation of a local community is relatively easy (Heino 2011; Boggero et al. 2014). Second, a set of multiple communities located within a drainage basin provides a good approximation of metacommunity limits because different drainage basins often harbor partly different biotas and unique environmental features to which organisms respond (Heino 2013; Henriques-Silva et al. 2013). Third, there is wide variation in several major biological and ecological traits among different freshwater organismal groups, ranging from bacteria through algae and macrophytes to animals (Heino et al. 2013; Verberk et al. 2013). Therefore, freshwater systems provide excellent opportunities for finding generalities, or lack of generalities, in factors correlating with emergent properties of metacommunities. They may also provide potential predictions about where, when, and in which settings a given metacommunity type should occur. Those settings could be revealed using correlates of metacommunity structure similar to biological traits of species in other contexts. Because the biological traits of species (Comont et al. 2012; Verberk et al. 2013) or, in our case, the ecological traits of metacommunities do not develop in isolation and are thus often correlated, we used composite trait variables as predictors of the elements of metacommunity structure and metacommunity types in our study.

We assembled a dataset of 45 freshwater metacommunities, ranging from temperate to Arctic drainage basins, from streams to lakes, and from bacteria to fish. Our aim was to (1) search for ecological correlates for the three elements (i.e., coherence, turnover, and boundary clumping) of metacommunity structure; (2) find out which metacommunity types prevail in freshwaters; and (3) examine which ecological and dataset characteristics separate observed metacommunity types. We hypothesized that the ecological characteristics of a metacommunity would be good predictors of variation in coherence, turnover, and boundary clumping because body size, trophic position, ecosystem type, and other traits may be related to the predictability of ecological patterns (Cottenie 2005; Soininen et al. 2007; De Bie et al. 2012). First (H_1_), we assumed that the distributions of small organisms are more stochastic than those of large organisms (Soininen et al. 2013), and metacommunities of small organisms should thus be more likely to exhibit randomness than those of large organisms, although opposite interpretations have also been suggested (De Bie et al. 2012). Second (H_2_), we hypothesized that metacommunity type should be related to the ecosystem type. For example, species in frequently disturbed lotic systems should show more individualistic responses to environmental gradients than species in more stable lentic systems. Gleasonian metacommunity types should thus prevail in lotic (Heino and Soininen 2005), whereas Clementsian metacommunity types should be more common in lentic systems (Henriques-Silva et al. 2013). Third (H_3_), the trophic position and life form of organisms should also be related to metacommunity type, although owing to lack of previous comparative studies we could not devise explicit hypotheses about which metacommunity types should be associated with a given trophic level and growth form. Fourth (H_4_), we assumed that increasing drainage basin area results in larger environmental heterogeneity and should thus promote high turnover (Heino 2011; Heino et al. 2015a), leading to Clementsian gradients. Fifth (H_5_), we assumed that latitude would be associated with metacommunity type because it is a proxy for climate conditions, which in turn should affect local habitat conditions and species distributions and result in geographical variation in metacommunity types (Henriques-Silva et al. 2013).

## Methods

### Datasets and metacommunity characteristics

We analyzed a dataset comprising 45 freshwater metacommunities (Appendix S1). We defined a metacommunity as a set of sites within a drainage basin, and thus, datasets crossing multiple drainage basins were not included. A local community was defined as a collection of organisms in a freshwater ecosystem (i.e., an entire lake for lentic–pelagic organisms, a stretch of littoral zone for lentic–benthic organisms, or a stream riffle site of about 100 m^2^ for lotic organisms). All the metacommunity datasets are from Finland (59°N to 70°N, 25°E to 32°E). Although the geographical area where those datasets come from is relatively small, we believe that comparative analyses based on high-quality datasets from a small region would provide more accurate information about metacommunities than more heterogeneous datasets assembled from various sources from over the world. Had we included some data from other continents, for example, we would also have to control for multiple large-scale factors (e.g., evolutionary factors) that are likely to generate differences in metacommunity organization. Furthermore, variation in the number of organismal groups was very high in our study area, and such versatility would perhaps have been difficult to obtain across large geographical areas. We had data for bacteria, algae (i.e., benthic diatoms, phytoplankton), macrophytes (i.e., vascular plants, bryophytes), invertebrates (i.e., benthic invertebrates, zooplankton), and vertebrates (i.e., fish).

We had a strict quality control for selecting each dataset. Each metacommunity dataset had to come from a single drainage basin and had to include at least 15 local communities, and all local communities had to have been sampled preferably in a relatively short period of time (i.e., typically within a single season in the same year). Only exceptions were the fish datasets which were collected using questionnaires, which aimed to reveal native fish species in the study lakes. The species list from a given metacommunity was carefully checked to guarantee that inconsistencies in identification were minimal.

Rather than relying on taxonomic delineations only, each of the 45 metacommunity datasets was also described by a number of organismal and ecosystem characteristics (Appendix S2). We first grouped the metacommunities by ecosystem type (lotic vs lentic). We also considered the average body size (continuous variable) of organisms comprising a metacommunity, broad trophic group (decomposer vs producer vs omnivore vs predator), life form (rooted vs benthic vs pelagic), and dispersal mode among localities (passive vs active). All those characteristics are approximations, referring to the ecologies of most species in a metacommunity. We did not use other organismal or ecosystem characteristics because those characteristics were either collinear with the traits we used or they proved to be less reliable ecologically. For example, owing to lack of strictly comparable data for the characteristics of all species in our data, we chose not to include a more comprehensive set of unreliable trait variables. This unreliability centers on the issue that we do not know for sure the trait variation within bacterial metacommunities, for example, and it is unlikely that any coarse measures for bacteria would be comparable to much better trait variables for fish. In addition, using drainage basin characteristics other than basin area would have been unfeasible because those characteristics are strongly related to the latitudinal location of a drainage basin.

### Analysis of the elements of metacommunity structure and metacommunity types

Elements of metacommunity structure (EMS) were assessed following Leibold and Mikkelson (2002) and Presley et al. (2010). We followed the “range perspective” in our analyses (Leibold and Mikkelson 2002). The EMS analysis is based on three metrics: coherence, turnover, and boundary clumping. Prior to calculating those metrics, site-by-species presence–absence matrices were ordinated using reciprocal averaging (i.e., correspondence analysis). Hence, the sites having similar species composition are close to each other and the species that have similar occurrence among the sites are close to each other along an ordination axis (Gauch 1982). Although correspondence analysis may be sensitive to rare species, we did not exclude them from the analyses for two reasons. First, most freshwater metacommunities in northern drainage basins are strongly dominated by rare species (bacteria: Heino et al. 2015b; diatoms: Soininen and Heino 2005; invertebrates: Heino 2005; bryophytes: Heino and Virtanen 2006; macrophytes: Alahuhta et al. 2014), so removing rare species would lead to unnatural results. Second, our previous analyses have shown that the main patterns found by the EMS analyses do not typically change if rare species (i.e., those occurring at a single site) are removed (Heino et al. 2015b).

*Coherence* is based on calculating the number of embedded absences (*Abs*) in the ordinated matrix and then comparing the observed value to a null distribution of embedded absences (i.e., gap in a species range) from simulated matrices (Leibold and Mikkelson 2002). A small number of embedded absences (i.e., *Abs* is significantly lower than expected by chance) suggest positive coherence, whereas a large number of embedded absences (i.e., *Abs* is significantly larger than expected by chance) suggest negative coherence. Significantly negative coherence thus suggests a checkerboard distribution of species (i.e., checkerboard metacommunity type), nonsignificant coherence refers to randomness (i.e., random metacommunity type), and significantly positive coherence is related to nestedness, evenly spaced gradients, Gleasonian gradients or Clementsian gradients (Leibold and Mikkelson 2002). *Turnover* is evaluated if coherence is positive, and it is measured as the number of times one species replaces (*Rep*) another between two sites in an ordinated matrix (Presley et al. 2010). Significant negative turnover (i.e., *Rep* is significantly lower than expected by chance) refers to nestedness (i.e., nested metacommunity type), whereas significantly positive turnover (i.e., *Rep* is significantly larger than expected by chance) indicates evenly spaced, Gleasonian or Clementsian metacommunity types (Leibold and Mikkelson 2002). The cases of significant positive coherence and nonsignificant turnover can be interpreted as quasi-structures (Presley et al. 2010). The evenly spaced, Gleasonian and Clementsian metacommunity types can be distinguished based on an index called boundary clumping (Leibold and Mikkelson 2002). Boundary clumping is analyzed using Morisita's dispersion index and a chi-square test comparing observed and expected distributions of range boundary locations. Values of Morisita's dispersion index that are not different from 1 indicate randomly distributed range boundaries (i.e., Gleasonian metacommunity type), values significantly larger than 1 indicate clumped range boundaries (i.e., Clementsian metacommunity type) and values significantly less than 1 indicate hyperdispersed range boundaries (i.e., evenly spaced metacommunity type). Correspondingly, quasi-evenly spaced, quasi-Gleasonian, and quasi-Clementsian metacommunity types can be separated by boundary clumping (Presley et al. 2010).

The significance of the index values for coherence (*Abs*) and turnover (*Rep*) was tested separately using the fixed-proportional null model, where row sums are fixed (i.e., the species richness of each site was maintained), but column marginal frequencies (i.e., species frequencies of occurrence) were used as probabilities. Random matrices were produced by the “r1” method for the fixed-proportional null model as implemented in the R package *vegan* (Oksanen et al. 2013). We also used the fixed–fixed null model (i.e., both species richness of each site and species frequencies are maintained) based on the “quasiswap” method in the R package vegan (Oksanen et al. 2013). We used 999 simulations to provide simulated matrices, with the exception of stream bacteria for which the very long computation time caused by very high numbers of species forced us to use 99 simulations. Statistical significance of *Abs* or *Rep* was then assessed by comparing the observed index value from the original matrix to the distribution of values derived from the randomizations (Manly 1995). Elements of metacommunity structure were evaluated for each metacommunity dataset based on axis 1 of reciprocal averaging because we were interested in the most important species compositional gradient. EMS analyses were done using the R package *metacom* (Dallas 2013) in the R environment (version 3.0.1, R Development Core Team 2013).

We also used a standardized effect size (SES) or a Z-score for the indices *Abs* and *Rep* for each dataset as (Gurevitch et al. 1992; Gotelli and McCabe 2002):

Z-score = (observed index value − mean index value based on simulations) / standard deviation of simulated index values.

Z-scores allow comparisons among datasets and can thus subsequently be used in comparative analyses. Basically, Z-scores between −1.96 and 1.96 are nonsignificant at *α* = 0.05 level and, thus, Z-scores of coherence and turnover can also be used to distinguish checkerboard, random, nested, and the remaining main three metacommunity types (Appendix S3). We also used the traditional approach to delineate metacommunity structures based on statistical significance (*P*-values) from the randomization tests of coherence and turnover (see above).

### Comparative analyses

We had nine predictor variables in the comparative analysis aimed to find correlates for explaining variation in the Z-scores of coherence, Z-scores of turnover or index of boundary clumping. We first used (1) number of sites; and (2) matrix fill (i.e., the proportion of “1s” in a presence–absence matrix) because dataset characteristics may have strong effects in comparative analyses of metacommunities (Heino et al. 2015c). We did not use the number of species as a predictor variable because it was significantly correlated with matrix fill (Spearman *r* = −0.412, *P* = 0.005) and because there was huge variation in and uneven distribution of the number of species among the metacommunity datasets (i.e., 12 to 6070). Second, we considered multiple ecological characteristics of a metacommunity as predictors, including average body size of organisms, trophic group (decomposer vs producer vs omnivore vs predator), ecosystem type (lentic vs lotic), life form (rooted vs benthic vs pelagic), and dispersal mode among localities (passive vs active). Many of the metacommunity traits are correlated. For example, pelagic organisms occur chiefly in lakes, and body size is often related to trophic level and dispersal mode (Rundle et al. 2007). We hence used Gower distance coefficient on the five metacommunity-level variables to produce a distance matrix across the 44 datasets (note that one metacommunity dataset was excluded here because it was an outlier in the comparison of the EMS analysis; see below) using function “daisy” in the R package *cluster* (Maechler et al. 2014). Gower distance coefficient allows using categorical variables, and we thus used that coefficient for calculating the distance matrix (Legendre and Legendre 2012). Thereafter, we ran a principal coordinates analysis (PCoA) on the Gower distance matrix to produce important components. We used the scores of each metacommunity along (3) PCoA1, (4) PCoA2, (5) PCoA3, and (6) PCoA4 components to indicate the combined ecological characteristics of a metacommunity. We also examined how beta diversity was related to the three elements of metacommunity structure. Hence, we partitioned total beta diversity (i.e., multiple site beta diversity based on Sørensen coefficient) in each metacommunity to beta diversity related to species compositional differences among sites (i.e., multiple site beta diversity based on Simpson coefficient) and nestedness resulting from species richness differences among sites using the function “beta.mul” in the R package *betapart* (Baselga and Orme 2012). We considered it important to use beta diversity as a predictor variable because it combines biological information about each metacommunity in a simple summary figure, although we acknowledge that it is inherently related to the metric “turnover” from the EMS analysis. We subsequently used (7) multiple site Simpson coefficient as predictor in the comparative analysis. Multiple site Simpson coefficient and multiple site nestedness coefficient were strongly negatively correlated (*r* = -0.895), and hence, collinearity problems precluded using both of them as predictors in the analyses. We used (8) total drainage basin area as proxy for environmental heterogeneity because it is a more useful variable than altitudinal range in a predominantly lowland region such as Finland. Finally, we used (9) latitude of a drainage basin as a predictor because geographical location and covarying climate variables may affect metacommunity patterns (Henriques-Silva et al. 2013).

We used generalized linear model (GLM) with Gaussian error as the method to analyze variation in the Z-scores of coherence, the Z-scores of turnover or the index of boundary clumping with all six variables described above as predictors. The variance inflation factors (VIF) of multiple site Simpson and multiple site nestedness indices were high (VIF > 10) in trial analyses, and hence, we used only multiple site Simpson index to avoid the problem of multicollinearity. Subsequently, the VIF values for the nine predictor variables were <4.3, indicating that there was no problem of collinearity among the predictor variables (Kutner et al. 2004). Had we used the original ecological categorical characteristics of the metacommunities instead of PCoA axes, we would also have ended up in multicollinearity problems because of nonindependent ecological characteristics. This would also have led a severe loss of degrees of freedom in our comparative analyses.

We also examined how well the nine predictor variables could distinguish observed metacommunity types using linear discriminant function analysis (DFA). Our response variable was categorical “metacommunity type”, and predictors were the nine continuous variables: number of sites, matrix fill, PCoA1, PCoA2, PCoA3, PCoA4, multiple site Simpson index, basin area, and latitude. DFA was conducted using the function “lda” in the R package *MASS* (Venables and Ripley 2002). We also used stepwise selection of predictor variables to see which predictors were most important in separating the metacommunity types using the function “greedy.wilks” in the R package *klaR* (Weihs et al. 2005). Finally, we used multivariate analysis of variance (MANOVA) to test for overall differences in the ecological characteristics among the metacommunity types.

## Results

**Figure 1:**
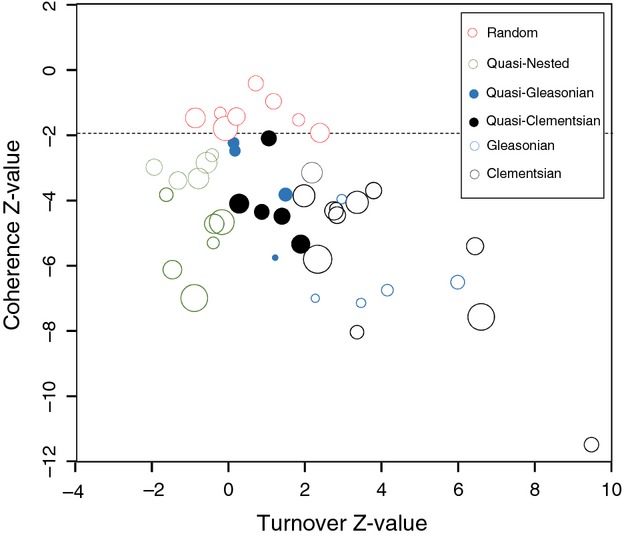
Metacommunity types of the 45 datasets plotted in the space of the Z-scores of coherence and turnover. Bubble size denotes the index of boundary clumping. A black open circle in the lower right corner indicates a metacommunity that was a clear outlier because of its very low coherence Z-value and very high turnover Z-value. It was thus excluded from the comparative analysis. Hence, the remaining 44 metacommunities were used in the comparative analysis. The dashed line indicates the coherence Z-score = −1.96.

The Z-scores of coherence from fixed-proportional (“r1”) or fixed–fixed (“quasiswap”) null models (*r* = 0.793, *P* < 0.001) were strongly correlated, and the same was true for the Z-scores of turnover (*r* = 0.907, *P* < 0001). Hence, we focused on the results based on the “r1” method because most previous studies have used it in the context of the EMS analysis. There was wide variation in the Z-scores of coherence, the Z-scores of turnover, and the index of boundary clumping across the 45 metacommunities (Appendix S4), resulting in six observed metacommunity types (Fig.1). We found that Clementsian (*n* = 12) and quasi-nested (*n* = 11) metacommunity types were most common, followed by random (*n* = 8), Gleasonian (*n* = 5), quasi-Clementsian (*n* = 5), and quasi-Gleasonian (*n* = 4) metacommunity types. Note that the same inferences can be drawn based on the p-values derived from randomization tests (Appendix S4). One metacommunity was an outlier with regard to coherence and turnover Z-values, and it was thus excluded from the subsequent comparative analyses of 44 metacommunities (Fig.1).

There was some variation among the five major organismal groups in the Z-scores of coherence (Kruskal–Wallis test, *χ*^2^ = 9.83, *P* = 0.043) and the measure of beta diversity related to nestedness (Kruskal–Wallis test, *χ*^2^ = 14.03, *P* = 0.007), but no significant differences were found among the organismal groups in the other four biological measures of metacommunities (Appendix S5). Furthermore, variations in the Z-scores of coherence, the Z-scores of turnover and the index of boundary clumping were weakly correlated (all *r* < 0.530) with the three measures of beta diversity (Appendix S6).

PCoA based on the Gower distance matrix of the ecological characteristics of metacommunities (i.e., body size, trophic group, ecosystem type, life form, and dispersal mode among sites) produced four principal coordinates with positive eigenvalues. PCoA1 (variance explained: 45.3%) showed variation from metacommunities of lotic–benthic producer organisms at the negative end of the axis to metacommunities of lentic–pelagic predator organisms at the positive end on the axis. PCoA2 (variance explained: 30.8%) showed variation from metacommunities of lentic–pelagic passively dispersing organisms at the negative end of the axis to metacommunities of lotic–benthic actively dispersing organisms at the positive end (Appendix S7). PCoA3 (variance explained: 17.4%) was mostly related to variation from metacommunities of benthic organisms to metacommunities of rooted plants. Along PCoA4 (variance explained: 5.9%), metacommunities varied from invertebrates at the negative end of the axis to bacteria at the positive end of the axis.

GLMs showed that no predictor variable was significantly associated with variation in the Z-scores of coherence (Table2). Simpson multiple site index was the only variable significantly related to the Z-scores of turnover. No predictor variable was significantly related to boundary clumping. This indicated that the single components of the EMS analysis are not necessarily strongly related to correlates describing metacommunity characteristics.

**Table 2:** GLM models for coherence Z-scores (a), turnover Z-scores (b), and boundary clumping index (c)

	Estimate	SE	*t*	*P*
(a) Coherence				
(Intercept)	2.211	9.392	0.235	0.815
No. Sites	−0.030	0.032	−0.914	0.367
Matrix fill	−5.092	6.483	−0.785	0.438
Simpson multiple	−3.272	5.827	−0.562	0.578
PCoA1	1.348	1.305	1.033	0.309
PCoA2	1.504	1.481	1.015	0.317
PCoA3	3.361	1.828	1.839	0.075
PCoA4	−5.710	2.966	−1.925	0.063
Basin area	−0.000	0.000	−1.621	0.114
Latitude	−0.011	0.121	−0.087	0.931
(b) Turnover				
(Intercept)	−26.682	8.400	−3.192	0.003
No. Sites	−0.009	0.029	−0.296	0.769
Matrix fill	10.007	5.798	1.738	0.091
Simpson multiple	16.663	5.211	3.191	**0.003**
PCoA1	−1.204	1.167	−1.029	0.309
PCoA2	0.963	1.325	0.727	0.472
PCoA3	−1.241	1.635	−0.759	0.453
PCoA4	0.269	2.653	0.102	0.919
Basin area	−0.000	0.000	−0.757	0.454
Latitude	0.202	0.108	1.873	0.069
(c) Boundary clumping				
(Intercept)	4.380	7.650	0.573	0.571
No. Sites	0.051	0.026	1.942	0.060
Matrix fill	−1.179	5.280	−0.223	0.825
Simpson multiple	−2.988	4.746	−0.630	0.533
PCoA1	0.509	1.063	0.479	0.635
PCoA2	−0.990	1.206	−0.821	0.418
PCoA3	−0.378	1.489	−0.254	0.801
PCoA4	1.932	2.416	0.800	0.429
Basin area	−0.000	0.000	−0.157	0.878
Latitude	0.006	0.098	−0.066	0.948

Significant effects are shown in bold font.

DFA with all nine predictor variables included showed that Clementsian, quasi-Clementsian, and quasi-nested metacommunity types were relatively well predicted to their original source groups, whereas Gleasonian, quasi-Gleasonian, and random metacommunity types were poorly predicted to the respective correct groups. The total classification success, 68.2%, was modest, but MANOVA showed that there was significant variation in the overall ecological characteristics among the metacommunity types (Wilks' lambda = 0.188, *F* = 1.610, *P* = 0.023). The DFA with stepwise selection of predictor variables showed that multiple site Simpson index and PCOA1 significantly discriminated between the observed metacommunity types, and MANOVA also showed significant differences in these two ecological characteristics among the metacommunity types (Wilks’ lambda = 0.476, *F* = 3.319, *P* = 0.001). However, this reduced model yielded a rather poor overall prediction success of 47.7%, with high correct predictions for Clementsian, Gleasonian, and quasi-nested metacommunity types, whereas the other metacommunity types were poorly predicted to correct groups (Table3).

**Table 3:** Summary of average values for the metacommunity characteristics. Also, shown are correct classifications (%) from discriminant function analysis based on the two significant predictors: Simpson multiple site beta diversity and the first metacommunity trait component (PCoA1)

Metacommunity type	No. Sites	Matrix fill	PCoA1	PCoA2	PCoA3	PCoA4	Simpson	Basin area	Latitude	Correct (%)
Clementsian	38	0.232	−0.006	0.021	0.029	0.007	0.872	25920	66.3	81.8
Gleasonian	18	0.214	−0.250	0.001	−0.092	0.021	0.827	26814	66.4	60.0
Quasi-Clementsian	19	0.290	−0.128	0.016	0.193	0.008	0.730	30508	64.2	20.0
Quasi-Gleasonian	20	0.238	−0.178	0.128	−0.013	−0.010	0.818	20145	65.5	0
Quasi-nested	27	0.260	0.231	−0.041	−0.053	0.006	0.747	28224	62.4	72.7
Random	20	0.219	0.015	−0.059	0.013	−0.031	0.798	19069	63.1	0

**Figure 2:**
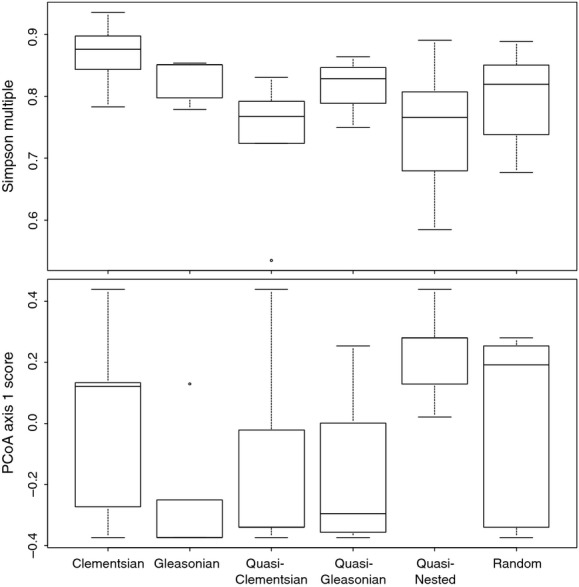
The six observed metacommunity types in relation to Simpson multiple site beta diversity and the PCoA axis 1.

Clementsian metacommunities showed highest and quasi-nested metacommunities lowest beta diversity based on Simpson multiple site index (Fig.2). Furthermore, Gleasonian metacommunities showed lowest scores along PCoA1, being metacommunities of lotic organisms, whereas quasi-nested metacommunities showed highest scores along PCoA1, being metacommunities of lentic organisms (Appendix S7). Finally, there was no contingency between the metacommunity types and the five major organismal groups (*χ*^2^-test with permutation, *χ*^2^ = 24.76, *P* = 0.213), suggesting that taxonomic group alone is a poor predictor of metacommunity type (Table4).

**Table 4:** Contingency table of taxonomic groups versus the six observed metacommunity types. *N* = 44 metacommunities. Q = quasi

Group	Clementsian	Gleasonian	Q-Clementsian	Q-Gleasonian	Q-Nested	Random	Total
Algae	4	4	1	1	2	2	14
Bacteria	1	0	0	0	4	1	6
Invertebrates	3	1	0	2	3	3	12
Macrophytes	2	0	3	1	0	2	8
Vertebrates	1	0	1	0	2	0	4
Total	11	5	5	4	11	8	**44**

## Discussion

Our comparative analyses showed that metacommunity structures vary widely in freshwater systems. We found that *the three elements of metacommunity structure* (i.e., coherence, turnover and boundary clumping) were weakly related to the predictor variables, which described dataset characteristics, ecological features of metacommunities, drainage basin area, and latitude. However, Simpson multiple site beta diversity was significantly positively related to turnover, which was obvious because both measure the same thing, that is, differences in species composition among sites (Leibold and Mikkelson 2002; Koleff et al. 2003). However, we pondered why the ecological characteristics of a metacommunity, drainage basin area, and latitude did not affect variation in the three elements of metacommunity structure. A reason to this lack of relationship may be that the factors underlying variation in *the three elements of metacommunity structure* should perhaps not be inferred too strongly in isolation, because it is their combined information which distinguishes different *metacommunity types* (Leibold & Mikkelson 2002; Presley et al. 2010). Despite this notion, the individual metrics are also useful, as indicated by the expected relationship between turnover and multiple site beta diversity.

We observed six *metacommunity types* following the classification proposed by Presley et al. (2010). Clementsian and quasi-nested metacommunity types prevailed in our study systems, whereas quasi-Clementsian, Gleasonian, and quasi-Gleasonian were less frequent. These findings cannot be easily associated with previous findings from our study area. For example, Heino and Soininen (2005) used a combination of different ordination methods, indices of nestedness, and indices of co-occurrence and found that the stream diatom dataset they analyzed showed multiple statistically significant structural patterns (e.g., there were more mutually exclusive pairs of species than expected by chance). Their interpretations were hampered by the absence of an objective means to choose which metacommunity pattern fitted best the empirical data. Furthermore, previous metacommunity studies in freshwater systems have frequently found significant nestedness (Heino et al. 2010; Soininen and Köngäs 2012) and significant negative co-occurrence (McCreadie and Bedwell 2013; Larsen and Ormerod 2014) for various organismal groups and ecosystem types. Hence, it was surprising that none of our metacommunities was associated with truly nested or checkerboard metacommunity types. An obvious reason to the differences between many earlier inferences and our present study is likely to be related to differences in statistical methodology and the fact that the EMS approach focuses on a single major gradient in the data, whereas various nestedness and co-occurrence indices examine patterns in the whole site-by-species matrix (Leibold and Mikkelson 2002; Presley et al. 2010). However, the EMS approach provides an objective means to assess the best fit of empirical data with metacommunity types because it compares several alternatives at the same time (Meynard et al. 2013; Dallas and Presley 2014).

Metacommunity studies utilizing the EMS approach have rarely been conducted in freshwater systems (Erős et al. 2014; Fernandes et al. 2014), and no study has compared bacteria, algae, macrophytes, invertebrates, and fish in the same comparative study. A previous study showed that there are geographical gradients in the prevalence of different metacommunity types of lake fish (Henriques-Silva et al. 2013). Henriques-Silva et al. (2013) found that Clementsian fish metacommunities prevailed in southern drainage basins in their Canadian study area, whereas nested metacommunities were more common in more northerly drainage basins. In our present study, variation in metacommunity types did not show a clear relationship with latitude, a finding which did not support our a priori hypothesis of geographical variation in metacommunity types. Instead, various metacommunity types occurred along the 1300 km latitudinal gradient in our study area. Along that latitudinal gradient, almost all climatic, vegetation, and geomorphological features vary strongly (Heino and Alahuhta 2015). Those environmental features affect regional species pools, drainage basin characteristics, and may eventually affect variation in metacommunity structuring (e.g., Heino et al. 2010). Alternatively, those features may make the situation unique for each dataset depending on the organismal group and underlying ecological conditions in a drainage basin (Heino et al. 2012; Soininen and Köngäs 2012). A different way of reasoning is also possible. For example, the fact that we included both lotic and lentic systems in our study may have decreased potential for generalizations in comparison with studies focused on a single ecosystem type only (cf. Henriques-Silva et al. 2013). In fact, in a related study where we analyzed data for bacteria, diatoms, bryophytes, and invertebrates surveyed at the same 70 stream sites in three drainage basins, we could observe clearer latitudinal patterns (Heino et al. 2015b). In that study, all organismal groups in the northernmost drainage basin (70°N) were associated with Clementsian metacommunity type, whereas Gleasonian and quasi-Gleasonian metacommunity types prevailed in the two southernmost drainage basins (66°N). However, even though there were similarities between those four groups of stream organisms in the geographical variation of metacommunity types, the underlying local environmental drivers varied among the organismal groups (Heino et al. 2015b).

It is also possible that ecological correlates other than latitude are more clearly associated with the observed metacommunity types. The most important ecological correlate related to variation in the metacommunity types was the first metacommunity trait component (i.e., PCoA axis 1), which portrayed variation from lotic–benthic producer metacommunities to lentic–pelagic predator metacommunities. This finding partly corroborated our second and third hypotheses that ecosystem type, life form, and trophic group of organisms are associated with metacommunity type. Decoupling the individual effects of those three features is difficult because they are intercorrelated. For example, pelagic organisms were absent in our stream datasets. Pelagic organisms are also generally less common in lotic than lentic systems. In contrast, we found little support for our first hypothesis about the relationship between randomness and body size (Soininen et al. 2013), although none of the four vertebrate datasets fitted best with random metacommunity type (cf. bacteria through algae and macrophytes to vertebrates; Table4). It is possible, however, that there is no linear relationship between body size and metacommunity type, but instead that the metacommunities of large organisms, such as fish, are less prone to show randomness than invertebrates, plants or microorganisms in freshwater systems. Our findings thus suggest some relationships among metacommunity types and their underlying ecological correlates, although one might expect even clearer patterns across so large variations in ecosystem types, life forms, and body sizes in our large set of metacommunities.

The metacommunity types best predicted to the correct type were Clementsian, Gleasonian, and quasi-nested metacommunity types. Clementsian and quasi-nested metacommunity types represent almost opposite ends with regard to species turnover among localities (Leibold & Mikkelson 2002; Presley and Willig 2010), and hence, it was not surprising that the levels of beta diversity differed between these metacommunity types. Furthermore, the ecological correlates of metacommunities also discriminated among these three metacommunity types, and especially Gleasonian and quasi-nested metacommunities seemed to differ in this respect. Gleasonian metacommunities were more likely to be represented by lotic organisms, while quasi-nested metacommunities were more likely to be composed of lentic organisms, including fingernail clam, snail, and fish (Appendix S7). This finding may be related to the fact that communities in island-like systems, such as lakes, may show ordered extinction–colonization dynamics that often underlie nested patterns along ecosystem size and isolation gradients (Wright et al. 1998). In contrast, communities in more continuous systems, such as streams, may show less ordered variations reflected by the individualistic responses of species to multiple environmental gradients (Heino 2013). Although our results do not provide absolutely clear picture of the relationships among beta diversity, metacommunity traits, and metacommunity types, they at least suggest that potential differences between those metacommunity types are worth additional studies.

Our results suggest that broad generalizations are possible to attain in community ecology, although many deterministic and stochastic factors are active simultaneously and affect local community structure (Lawton 1999) and metacommunity organization (Leibold et al. 2004). We presumed that patterns emerging at the level of entire metacommunities would disregard the local-scale contingencies and lead to patterns that are more easily interpretable. However, our present results also emphasize the need to admit the potential complexity of inferences in community ecology. Hence, community ecologists should also focus on factors responsible for causing context dependency, such as (1) different responses of different organismal groups to ecological gradients in the same drainage basin (Beisner et al. 2006); (2) the responses of the same organismal group to different ecological gradients in different drainage basins (Henriques-Silva et al. 2013); and (3) differences in the responses of the same organismal group to the physiographic templates among major ecosystem types (e.g., streams versus lakes). Our perception may also be weakened by the fact that the metacommunity patterns of the same organismal group based on sampling the same set of sites may vary in time (Erős et al. 2014; Fernandes et al. 2014). We acknowledge that testing these ideas more directly would have entailed inclusion of directly comparable information about the environmental conditions of all sites in each metacommunity (Heino et al. 2015b). However, this would have been highly challenging for such versatility of organisms, ecosystems, and drainage basins we compared in this study. To this end, we emphasize that all metacommunity types are worth consideration in ecological studies, and that multiple explanations are likely as to the structuring of local communities and metacommunities.

## Data Availability

The data for metacommunity traits and the results from a large number of EMS analyses are shown in Supporting Information (Appendices S1–S4).

## Supporting Information

**Appendix S1.** The metacommunity datasets used in this paper. **Appendix S2**. The characteristics of the metacommunity datasets used in the comparative analysis. **Appendix S3.** Inferring metacommunity types. **Appendix S4.** The EMS results for each dataset. **Appendix S5.** Variation among the main five taxonomic groups in metacommunity metrics. **Appendix S6.** Correlations between the three elements of metacommunity structure. **Appendix S7.** The 44 metacommunities plotted in the “metacommunity characteristics space” of Principal Coordinates Analysis.
