# Supplementary material for: A comparative analysis of metacommunity types in the freshwater realm
Source: Ecol Evol. 2015 Mar 11;5(7):1525–37. doi: 10.1002/ece3.1460 (PMC4395181; doi:10.1002/ece3.1460)
Supplement: Supplementary file 1 — Appendix S1. The metacommunity datasets used in this paper. Appendix S2. The characteristics of the metacommunity datasets used in the comparative analysis. Appendix S3. Inferring metacommunity types. Appendix S4. The EMS results for each dataset. Appendix S5. Variation among the main five taxonomic groups in metacommunity metrics. Appendix S6. Correlations between the three elements of metacommunity structure. Appendix S7. The 44 metacommunities plotted in the “metacommunity characteristics space” of Principal Coordinates Analysis. [file ece30005-1525-sd1.docx]

**Supporting information**

Heino, J., Soininen, J., Alahuhta, J., Lappalainen, J & Virtanen, R. A comparative analysis of metacommunity types in the freshwater realm. *Ecology and Evolution*.

**Appendix S1.** The metacommunity datasets used in this paper

**Appendix S2**. The characteristics of the metacommunity datasets used in the comparative analysis.

**Appendix S3.** Inferring metacommunity types based on Z-scores.

**Appendix S4.** The EMS results for each dataset.

**Appendix 5.** Variation among the main five taxonomic groups in metacommunity metrics.

**Appendix 6.** Correlations between the three elements of metacommunity structure.

**Appendix 7.** The 44 metacommunities plotted in the “metacommunity characteristics space” of Principal Coordinates Analysis.

Appendix S1. The metacommunity datasets used in this paper. Also, shown are the publications where the whole data or part of the data have been used previously.

| Dataset abbreviation | Original article |
| --- | --- |
| Stream_dia_Iijoki | Heino, J. et al. (2012) Context dependency and metacommunity structuring in boreal headwater streams. Oikos 121: 537-544 |
| Stream_bry_Iijoki | Heino, J. et al. (2012) Context dependency and metacommunity structuring in boreal headwater streams. Oikos 121: 537-544 |
| Stream_inv_Iijoki | Heino, J. et al. (2012) Context dependency and metacommunity structuring in boreal headwater streams. Oikos 121: 537-544 |
| Stream_dia_Kouta | Heino, J. et al. (2012) Context dependency and metacommunity structuring in boreal headwater streams. Oikos 121: 537-544 |
| Stream_bry_Kouta | Heino, J. et al. (2012) Context dependency and metacommunity structuring in boreal headwater streams. Oikos 121: 537-544 |
| Stream_inv_Kouta | Heino, J. et al. (2012) Context dependency and metacommunity structuring in boreal headwater streams. Oikos 121: 537-544 |
| Stream_bry_Kuusa | Virtanen, R. (unpublished) |
| Stream_dia_Teno | Heino, J. et al. (2015b) Elements of metacommunity structure and community-environment relationships in stream organisms. Freshwater Biology, in press. |
| Stream_bac_Teno | Heino, J. et al. (2015b) Elements of metacommunity structure and community-environment relationships in stream organisms. Freshwater Biology, in press. |
| Stream_inv_Teno | Heino, J. et al. (2015b) Elements of metacommunity structure and community-environment relationships in stream organisms. Freshwater Biology, in press. |
| Lake_maph_Kymi | Alahuhta, J. (unpublished) |
| Lake_maph_Vuoksi | Alahuhta, J. (unpublished) |
| Lake_fish_area14 | Lappalainen, J. & Malinen, T. (2002) Effects of area and location on pikeperch yields in Finnish la­kes. In: Cowx I. G. (ed.) Management and Ecology of Lake and River Fisheries. pp. 35-45. |
| Lake_fish_area04 | Lappalainen, J. & Malinen, T. (2002) Effects of area and location on pikeperch yields in Finnish la­kes. In: Cowx I. G. (ed.) Management and Ecology of Lake and River Fisheries. pp. 35-45.  Kekäläinen et al. (2007) Fish community data in determining the ecological status of humic lakes (In Finnish). Reports of the North Karelia Regional Environment Centre 5 / 2007. 46 pp. |
| Lake_fish_area35 | Lappalainen, J. & Malinen, T. (2002) Effects of area and location on pikeperch yields in Finnish la­kes. In: Cowx I. G. (ed.) Management and Ecology of Lake and River Fisheries. pp. 35-45. |
| Lake_fish_area59 | Lappalainen, J. & Malinen, T. (2002) Effects of area and location on pikeperch yields in Finnish la­kes. In: Cowx I. G. (ed.) Management and Ecology of Lake and River Fisheries. pp. 35-45.  Salojärvi, K., & Ekholm, P. (1990). Predicting the efficiency of whitefish (*Coregonus lavaretus* L. sl) stocking from pre-stocking catch statistics. In: *Management of freshwater fisheries: proceedings of a symposium organized by the European Inland Fisheries Advisory Commission, Göteborg, Sweden, 31 May-3 June 1988*. Centre for Agricultural Publishing and Documentation (Pudoc). |
| Lake_clam_Pirkan | Aho, J. (1966) Ecological basis of the distribution of the littoral freshwater molluscs in the vicinity of Tampere, South Finland. Annales Zoologici Fennici 3: 287–322. |
| Lake_snail_Pirkan | Aho, J. (1966) Ecological basis of the distribution of the littoral freshwater molluscs in the vicinity of Tampere, South Finland. Annales Zoologici Fennici 3: 287–322. |
| Lake_inv_Oulanka | Heino, J. (2013) Does dispersal ability affect the relative importance of environmental control and spatial structuring of littoral macroinvertebrate communities? Oecologia 171: 971-980. |
| Pond_zpl_Kouta | Soininen, J. et al. (2007) Neutrality, niches and determinants of plankton metacommunity structure across boreal wetland ponds. Ecoscience 14: 146-154. |
| Pond_kapl_Kouta | Soininen, J. et al. (2007) Neutrality, niches and determinants of plankton metacommunity structure across boreal wetland ponds. Ecoscience 14: 146-154. |
| Lake_zpl_Evo | Soininen, J. et al. (2011) Disentangling the spatial patterns in community composition of prokaryotic and eukaryotic lake plankton. Limnology & Oceanography 56: 508-520. |
| Lake_zpl_Konne | Soininen, J. et al. (2011) Disentangling the spatial patterns in community composition of prokaryotic and eukaryotic lake plankton. Limnology & Oceanography 56: 508-520. |
| Lake_zpl_Kuusa | Soininen, J. et al. (2011) Disentangling the spatial patterns in community composition of prokaryotic and eukaryotic lake plankton. Limnology & Oceanography 56: 508-520. |
| Lake_zpl_Tvar | Soininen, J. et al. (2011) Disentangling the spatial patterns in community composition of prokaryotic and eukaryotic lake plankton. Limnology & Oceanography 56: 508-520. |
| Lake_zpl_Vantaa | Soininen, J. et al. (2011) Disentangling the spatial patterns in community composition of prokaryotic and eukaryotic lake plankton. Limnology & Oceanography 56: 508-520. |
| Lake_bac_Evo | Soininen, J. et al. (2011) Disentangling the spatial patterns in community composition of prokaryotic and eukaryotic lake plankton. Limnology & Oceanography 56: 508-520. |
| Lake_bac_Konne | Soininen, J. et al. (2011) Disentangling the spatial patterns in community composition of prokaryotic and eukaryotic lake plankton. Limnology & Oceanography 56: 508-520. |
| Lake_bac_Kuusa | Soininen, J. et al. (2011) Disentangling the spatial patterns in community composition of prokaryotic and eukaryotic lake plankton. Limnology & Oceanography 56: 508-520. |
| Lake_bac_Tvar | Soininen, J. et al. (2011) Disentangling the spatial patterns in community composition of prokaryotic and eukaryotic lake plankton. Limnology & Oceanography 56: 508-520. |
| Lake_bac_Vantaa | Soininen, J. et al. (2011) Disentangling the spatial patterns in community composition of prokaryotic and eukaryotic lake plankton. Limnology & Oceanography 56: 508-520. |
| Lake_kapl_Evo | Soininen, J. et al. (2011) Disentangling the spatial patterns in community composition of prokaryotic and eukaryotic lake plankton. Limnology & Oceanography 56: 508-520. |
| Lake_kapl_Konne | Soininen, J. et al. (2011) Disentangling the spatial patterns in community composition of prokaryotic and eukaryotic lake plankton. Limnology & Oceanography 56: 508-520. |
| Lake_kapl_Kuusa | Soininen, J. et al. (2011) Disentangling the spatial patterns in community composition of prokaryotic and eukaryotic lake plankton. Limnology & Oceanography 56: 508-520. |
| Lake_kapl_Tvar | Soininen, J. et al. (2011) Disentangling the spatial patterns in community composition of prokaryotic and eukaryotic lake plankton. Limnology & Oceanography 56: 508-520. |
| Lake_kapl_Vantaa | Soininen, J. et al. (2011) Disentangling the spatial patterns in community composition of prokaryotic and eukaryotic lake plankton. Limnology & Oceanography 56: 508-520. |
| Pond_dia_Kilpis | Soininen, J. (unpublished) |
| Lake_dia_Inari* | Soininen, J. (unpublished) |
| Stream_dia_Kemij | Soininen, J. et al. (2009) Local-regional diversity relationship varies with spatial scale in lotic diatoms. Journal of Biogeography 36: 720-727. |
| Stream_dia_Muonio | Soininen, J. et al. (2009) Local-regional diversity relationship varies with spatial scale in lotic diatoms. Journal of Biogeography 36: 720-727. |
| Stream_dia_Utsjoki | Soininen, J. et al. (2009) Local-regional diversity relationship varies with spatial scale in lotic diatoms. Journal of Biogeography 36: 720-727. |
| Stream_dia_Vantaa | Soininen, J. et al. (2009) Local-regional diversity relationship varies with spatial scale in lotic diatoms. Journal of Biogeography 36: 720-727. |
| Stream_bry_Kemij | Virtanen, R. (unpublished) |
| Stream_bry_Muonio | Virtanen, R. (unpublished) |
| Stream_bry_Utsj | Virtanen, R. (unpublished) |

* An outlier (coherence Z and turnover Z) dataset that was not include in the comparative analysis.

Appendix S2. The characteristics of the metacommunity datasets used in the comparative analysis. Abbreviations: Coh Z = Z-value of coherence; Turn Z = Z-value of turnover; Bound Index = Boundary index; Sor = multiple site Sørensen dissimilarity; Sim = multiple site Simpson dissimilarity; Nes = multiple site dissimilarity resulting from nestedness. Area = total area of a drainage basin (km^2^).

| Dataset | Basin name | No. Sites | No. Species | Matrix fill | Sor | Sim | Nes | Body size | Trophic  group | Ecosystem  type | Life form | Dispersal  mode | Group | Area | Latitude |
| --- | --- | --- | --- | --- | --- | --- | --- | --- | --- | --- | --- | --- | --- | --- | --- |
| Stream_dia_Iijoki | Iijoki | 20 | 170 | 0.24 | 0.87 | 0.80 | 0.07 | -8.11 | prod | lotic | bent | pass | alg | 14191 | 65 |
| Stream_bry_Iijoki | Iijoki | 19 | 21 | 0.32 | 0.85 | 0.75 | 0.09 | 1 | prod | lotic | rooted | pass | mp | 14191 | 65 |
| Stream_inv_Iijoki | Iijoki | 20 | 149 | 0.25 | 0.87 | 0.83 | 0.04 | -1.58 | omni | lotic | bent | acti | inv | 14191 | 65 |
| Stream_dia_Kouta | Koutajoki | 20 | 182 | 0.2 | 0.89 | 0.83 | 0.06 | -8.11 | prod | lotic | bent | pass | alg | 26100 | 66 |
| Stream_bry_Kouta | Koutajoki | 20 | 40 | 0.17 | 0.91 | 0.83 | 0.08 | 1 | prod | lotic | rooted | pass | mp | 26100 | 66 |
| Stream_inv_Kouta | Koutajoki | 20 | 164 | 0.22 | 0.89 | 0.85 | 0.03 | -1.58 | omni | lotic | bent | acti | inv | 26100 | 66 |
| Stream_bry_Kuusa | Koutajoki | 47 | 29 | 0.2 | 0.92 | 0.88 | 0.04 | 1 | prod | lotic | rooted | pass | mp | 26100 | 66 |
| Stream_dia_Teno | Tenojoki | 28 | 118 | 0.24 | 0.89 | 0.84 | 0.05 | -8.11 | prod | lotic | bent | pass | alg | 16374 | 70 |
| Stream_bac_Teno | Tenojoki | 24 | 6070 | 0.09 | 0.95 | 0.93 | 0.01 | -10 | deco | lotic | bent | pass | bac | 16374 | 70 |
| Stream_inv_Teno | Tenojoki | 30 | 98 | 0.21 | 0.9 | 0.85 | 0.05 | -1.58 | omni | lotic | bent | acti | inv | 16374 | 70 |
| Lake_maph_Kymi | Kymijoki | 45 | 98 | 0.32 | 0.92 | 0.87 | 0.05 | 1 | prod | lentic | rooted | pass | mp | 37107 | 62 |
| Lake_maph_Vuoksi | Vuoksi | 26 | 87 | 0.26 | 0.85 | 0.79 | 0.06 | 1 | prod | lentic | rooted | pass | mp | 61560 | 62 |
| Lake_fish_area14 | Kymijoki | 29 | 23 | 0.41 | 0.79 | 0.59 | 0.20 | 2.43 | pred | lentic | pela | acti | ver | 37107 | 62 |
| Lake_fish_area04 | Vuoksi | 84 | 30 | 0.4 | 0.92 | 0.8 | 0.12 | 2.43 | pred | lentic | pela | acti | ver | 61560 | 62 |
| Lake_fish_area35 | Kokemäenjoki | 86 | 28 | 0.43 | 0.93 | 0.88 | 0.10 | 2.43 | pred | lentic | pela | acti | ver | 27100 | 62 |
| Lake_fish_area59 | Oulujoki | 20 | 25 | 0.48 | 0.72 | 0.54 | 0.19 | 2.43 | pred | lentic | pela | acti | ver | 22900 | 65 |
| Lake_clam_Pirkan | Kokemäenjoki | 21 | 12 | 0.34 | 0.85 | 0.59 | 0.26 | -1.58 | omni | lentic | bent | pass | inv | 27100 | 62 |
| Lake_snail_Pirkan | Kokemäenjoki | 19 | 18 | 0.34 | 0.85 | 0.63 | 0.22 | -1.6 | omni | lentic | bent | pass | inv | 27100 | 62 |
| Lake_inv_Oulanka | Koutajoki | 48 | 155 | 0.22 | 0.94 | 0.91 | 0.02 | -1.58 | omni | lentic | bent | acti | inv | 26100 | 66 |
| Pond_zpl_Kouta | Koutajoki | 25 | 65 | 0.38 | 0.84 | 0.78 | 0.06 | -3.74 | omni | lentic | pela | pass | inv | 26100 | 66 |
| Pond_kapl_Kouta | Koutajoki | 25 | 125 | 0.14 | 0.92 | 0.89 | 0.03 | -9.41 | prod | lentic | pela | pass | alg | 26100 | 66 |
| Lake_zpl_Evo | Kokemäenjoki | 20 | 34 | 0.26 | 0.87 | 0.82 | 0.05 | -3.74 | omni | lentic | pela | pass | inv | 27100 | 62 |
| Lake_zpl_Konne | Kymijoki | 20 | 31 | 0.31 | 0.82 | 0.73 | 0.09 | -3.74 | omni | lentic | pela | pass | inv | 37107 | 63 |
| Lake_zpl_Kuusa | Koutajoki | 20 | 36 | 0.18 | 0.9 | 0.86 | 0.04 | -3.74 | omni | lentic | pela | pass | inv | 26100 | 66 |
| Lake_zpl_Tvar | Karjaanjoki | 20 | 46 | 0.21 | 0.89 | 0.85 | 0.03 | -3.74 | omni | lentic | pela | pass | inv | 2045 | 59 |
| Lake_zpl_Vantaa | Vantaanjoki | 20 | 33 | 0.26 | 0.87 | 0.83 | 0.05 | -3.74 | omni | lentic | pela | pass | inv | 1680 | 60 |
| Lake_bac_Evo | Kokemäenjoki | 20 | 46 | 0.18 | 0.87 | 0.76 | 0.11 | -10 | deco | lentic | pela | pass | bac | 27100 | 62 |
| Lake_bac_Konne | Kymijoki | 20 | 41 | 0.21 | 0.86 | 0.77 | 0.09 | -10 | deco | lentic | pela | pass | bac | 37107 | 63 |
| Lake_bac_Kuusa | Koutajoki | 20 | 50 | 0.17 | 0.89 | 0.81 | 0.07 | -10 | deco | lentic | pela | pass | bac | 26100 | 66 |
| Lake_bac_Tvar | Karjaanjoki | 20 | 59 | 0.18 | 0.89 | 0.80 | 0.08 | -10 | deco | lentic | pela | pass | bac | 2045 | 59 |
| Lake_bac_Vantaa | Vantaanjoki | 20 | 33 | 0.19 | 0.87 | 0.69 | 0.18 | -10 | deco | lentic | pela | pass | bac | 1680 | 60 |
| Lake_kapl_Evo | Kokemäenjoki | 20 | 89 | 0.2 | 0.89 | 0.83 | 0.05 | -9.41 | prod | lentic | pela | pass | alg | 27100 | 62 |
| Lake_kapl_Konne | Kymijoki | 20 | 133 | 0.19 | 0.89 | 0.85 | 0.04 | -9.41 | prod | lentic | pela | pass | alg | 37107 | 63 |
| Lake_kapl_Kuusa | Koutajoki | 20 | 103 | 0.16 | 0.9 | 0.88 | 0.03 | -9.41 | prod | lentic | pela | pass | alg | 26100 | 66 |
| Lake_kapl_Tvar | Karjaanjoki | 20 | 95 | 0.18 | 0.89 | 0.86 | 0.03 | -9.41 | prod | lentic | pela | pass | alg | 2045 | 59 |
| Lake_kapl_Vantaa | Vantaanjoki | 20 | 136 | 0.19 | 0.89 | 0.85 | 0.04 | -9.41 | prod | lentic | pela | pass | alg | 1680 | 60 |
| Pond_dia_Kilpis | Tornionjoki | 44 | 199 | 0.1 | 0.95 | 0.94 | 0.02 | -8.11 | prod | lentic | pela | pass | alg | 40300 | 69 |
| Lake_dia_Inari* | Paatsjoki | 45 | 125 | 0.37 | 0.92 | 0.89 | 0.03 | -8.11 | prod | lentic | bent | pass | alg | 14512 | 69 |
| Stream_dia_Kemij | Kemijoki | 15 | 164 | 0.26 | 0.84 | 0.79 | 0.05 | -8.11 | prod | lotic | bent | pass | alg | 51000 | 67 |
| Stream_dia_Muonio | Tornionjoki | 15 | 206 | 0.24 | 0.84 | 0.78 | 0.06 | -8.11 | prod | lotic | bent | pass | alg | 40300 | 68 |
| Stream_dia_Utsjoki | Tenojoki | 15 | 144 | 0.18 | 0.89 | 0.85 | 0.03 | -8.11 | prod | lotic | bent | pass | alg | 16374 | 70 |
| Stream_dia_Vantaa | Vantaanjoki | 15 | 118 | 0.33 | 0.79 | 0.72 | 0.06 | -8.11 | prod | lotic | bent | pass | alg | 1680 | 60 |
| Stream_bry_Kemij | Kemijoki | 31 | 32 | 0.14 | 0.93 | 0.89 | 0.05 | 1 | prod | lotic | rooted | pass | mp | 51000 | 67 |
| Stream_bry_Muonio | Tornionjoki | 15 | 37 | 0.21 | 0.86 | 0.77 | 0.09 | 1 | prod | lotic | rooted | pass | mp | 40300 | 68 |
| Stream_bry_Utsj | Tenojoki | 15 | 44 | 0.24 | 0.85 | 0.68 | 0.17 | 1 | prod | lotic | rooted | pass | mp | 16374 | 70 |

* An outlier (coherence Z-score and turnover Z-score) dataset that was not include in the comparative analysis.

Appendix S3. Inferring metacommunity types using the Z-scores of coherence and the Z-scores of turnover. Morisita index is used for inferring boundary clumping. Z-scores between -1.96 and 1.96 are non-significant at α= 0.05. See manuscript text for details.

Appendix S4. The EMS results for each dataset. These results were based on the fixed-proportional (r1) null model and the first reciprocal averaging axis. The interpretation follows Presley *et al*. (2010). Abbreviations: Coh Z = Z-value of coherence; Tur Z = Z-value of turnover; Q = Quasi.

| Dataset | Coherence | |  |  |  | Turnover |  |  |  |  | Boundary clumping | |  |  |
| --- | --- | --- | --- | --- | --- | --- | --- | --- | --- | --- | --- | --- | --- | --- |
|  | Abs | Coh Z | p | Sim mean | Sim sd | Rep | Tur Z | p | Sim mean | Sim sd | Index | p | df | Interpretation |
| Stream_dia_Iijoki | 1361 | -7.10 | 0.001 | 1979 | 87 | 77330 | 3.47 | 0.001 | 35621 | 12035 | 0.72 | 0.291 | 167 | Gleasonian |
| Stream_bry_Iijoki | 108 | -2.44 | 0.014 | 148 | 17 | 1678 | 0.17 | 0.864 | 1590 | 513 | 1.00 | 0.533 | 18 | Q-Gleasonian |
| Stream_inv_Iijoki | 1416 | -3.78 | 0.001 | 1683 | 71 | 42006 | 1.49 | 0.136 | 28723 | 8913 | 1.46 | 0.157 | 146 | Q-Gleasonian |
| Stream_dia_Kouta | 1620 | -5.72 | 0.001 | 2221 | 105 | 67199 | 1.22 | 0.221 | 47667 | 15972 | 0.26 | 0.066 | 179 | Q-Gleasonian |
| Stream_bry_Kouta | 259 | -2.06 | 0.039 | 320 | 29 | 8906 | 1.06 | 0.291 | 6593 | 2188 | 2.00 | 0.001 | 37 | Q-Clementsian |
| Stream_inv_Kouta | 1411 | -6.47 | 0.001 | 1946 | 83 | 100784 | 5.99 | 0.001 | 35454 | 10909 | 1.61 | 0.106 | 161 | Gleasonian |
| Stream_bry_Kuusa | 522 | -4.22 | 0.001 | 684 | 38 | 27030 | 2.81 | 0.004 | 15165 | 4216 | 1.46 | 0.013 | 44 | Clementsian |
| Stream_dia_Teno | 1509 | -4.41 | 0.001 | 1872 | 83 | 69452 | 2.84 | 0.004 | 35317 | 12027 | 2.35 | 0.001 | 115 | Clementsian |
| Stream_bac_Teno | 89452 | -7.53 | 0.001 | 117930 | 3780 | 75769955 | 6.60 | 0.001 | 14761506 | 9232680 | 5.86 | 0.024 | 6067 | Clementsian |
| Stream_inv_Teno | 1396 | -3.11 | 0.002 | 1655 | 83 | 60240 | 2.18 | 0.029 | 34073 | 11998 | 3.60 | 0.001 | 95 | Clementsian |
| Lake_maph_Kymi | 1858 | -5.76 | 0.001 | 2452 | 103 | 82710 | 2.33 | 0.019 | 46293 | 15620 | 6.65 | 0.001 | 95 | Clementsian |
| Lake_maph_Vuoksi | 907 | -4.44 | 0.001 | 1129 | 50 | 21769 | 1.40 | 0.161 | 15225 | 4671 | 2.15 | 0.001 | 84 | Q-Clementsian |
| Lake_fish_area14 | 110 | -6.08 | 0.001 | 205 | 16 | 524 | -1.46 | 0.140 | 1223 | 478 | 2.89 | 0.001 | 20 | Q-nested |
| Lake_fish_area04 | 605 | -6.65 | 0.001 | 986 | 57 | 8818 | -0.80 | 0.420 | 12831 | 4991 | 6.06 | 0.001 | 27 | Q-nested |
| Lake_fish_area35 | 773 | -4.02 | 0.001 | 993 | 54 | 24513 | 3.36 | 0.001 | 10685 | 4109 | 4.21 | 0.001 | 25 | Clementsian |
| Lake_fish_area59 | 86 | -4.06 | 0.001 | 152 | 16 | 1286 | 0.28 | 0.770 | 1151 | 406 | 3.03 | 0.001 | 22 | Q-Clementsian |
| Lake_clam_Pirkan | 40 | -2.94 | 0.003 | 69 | 10 | 124 | -1.94 | 0.052 | 603 | 247 | 2.22 | 0.001 | 9 | Q-nested |
| Lake_snail_Pirkan | 78 | -2.80 | 0.005 | 109 | 11 | 1013 | -0.57 | 0.568 | 1228 | 377 | 3.70 | 0.001 | 15 | Q-nested |
| Lake_inv_Oulanka | 3097 | -5.36 | 0.001 | 4652 | 138 | 301920 | 6.44 | 0.001 | 96857 | 31864 | 2.49 | 0.001 | 152 | Clementsian |
| Pond_zpl_Kouta | 585 | -3.66 | 0.001 | 721 | 37 | 16915 | 3.79 | 0.001 | 7734 | 2422 | 2.23 | 0.001 | 62 | Clementsian |
| Pond_kapl_Kouta | 1863 | -4.62 | 0.001 | 2363 | 108 | 62528 | -0.17 | 0.861 | 66418 | 22328 | 5.19 | 0.001 | 149 | Q-nested |
| Lake_zpl_Evo | 259 | -1.29 | 0.198 | 291 | 25 | 4082 | -0.21 | 0.830 | 4370 | 1349 | 1.19 | 0.001 | 31 | Random |
| Lake_zpl_Konne | 185 | -2.58 | 0.009 | 247 | 24 | 2417 | -0.43 | 0.668 | 2810 | 918 | 1.44 | 0.035 | 28 | Q-nested |
| Lake_zpl_Kuusa | 241 | -2.20 | 0.028 | 303 | 28 | 7346 | 0.14 | 0.890 | 7091 | 1846 | 1.07 | 0.347 | 33 | Q-Gleasonian |
| Lake_zpl_Tvar | 423 | -0.37 | 0.710 | 434 | 31 | 9979 | 0.71 | 0.474 | 8305 | 2341 | 1.94 | 0.001 | 43 | Random |
| Lake_zpl_Vantaa | 250 | -1.49 | 0.136 | 284 | 23 | 6866 | 1.83 | 0.066 | 4504 | 1287 | 1.30 | 0.088 | 30 | Random |
| Lake_bac_Evo | 209 | -4.68 | 0.001 | 415 | 44 | 7832 | -0.37 | 0.711 | 8936 | 2990 | 3.17 | 0.001 | 43 | Quasi-nested |
| Lake_bac_Konne | 242 | -3.28 | 0.001 | 358 | 35 | 4855 | -0.78 | 0.435 | 6519 | 2131 | 3.61 | 0.001 | 38 | Quasi-nested |
| Lake_bac_Kuusa | 317 | -3.78 | 0.001 | 467 | 39 | 5676 | -1.62 | 0.105 | 11151 | 3380 | 1.57 | 0.023 | 47 | Quasi-nested |
| Lake_bac_Tvar | 422 | -3.35 | 0.001 | 574 | 45 | 7824 | -1.31 | 0.188 | 13334 | 4193 | 2.59 | 0.001 | 56 | Quasi-nested |
| Lake_bac_Vantaa | 219 | -1.43 | 0.152 | 261 | 30 | 4090 | -0.86 | 0.388 | 5559 | 1703 | 3.31 | 0.001 | 30 | Random |
| Lake_kapl_Evo | 727 | -4.27 | 0.001 | 966 | 56 | 34624 | 2.76 | 0.005 | 18413 | 5879 | 2.85 | 0.001 | 86 | Clementsian |
| Lake_kapl_Konne | 1043 | -6.71 | 0.001 | 1557 | 76 | 74827 | 4.15 | 0.001 | 32980 | 10076 | 1.15 | 0.131 | 130 | Gleasonian |
| Lake_kapl_Kuusa | 923 | -3.82 | 0.001 | 1178 | 67 | 45510 | 1.97 | 0.048 | 27740 | 9010 | 4.02 | 0.001 | 100 | Clementsian |
| Lake_kapl_Tvar | 756 | -5.27 | 0.001 | 1065 | 58 | 18426 | -0.40 | 0.691 | 21104 | 6751 | 1.19 | 0.015 | 92 | Q-nested |
| Lake_kapl_Vantaa | 1449 | -1.76 | 0.079 | 1585 | 77 | 31960 | -0.07 | 0.941 | 32793 | 11211 | 4.96 | 0.001 | 133 | Random |
| Pond_dia_Kilpis | 4074 | -8.00 | 0.001 | 5820 | 218 | 596992 | 3.36 | 0.001 | 272520 | 96409 | 1.56 | 0.013 | 196 | Clementsian |
| Lake_dia_Inari* | 2398 | -11.45 | 0.001 | 3095 | 60 | 98112 | 9.48 | 0.001 | 21506 | 8079 | 1.79 | 0.001 | 122 | Clementsian |
| Stream_dia_Kemij | 1257 | -1.39 | 0.163 | 1344 | 62 | 23985 | 0.21 | 0.831 | 22502 | 6980 | 2.57 | 0.013 | 161 | Random |
| Stream_dia_Muonio | 1165 | -6.96 | 0.001 | 1770 | 87 | 58149 | 2.27 | 0.022 | 33957 | 10617 | 0.53 | 0.275 | 203 | Gleasonian |
| Stream_dia_Utsjoki | 994 | -3.92 | 0.001 | 1253 | 66 | 52771 | 2.96 | 0.002 | 26484 | 8856 | 0.75 | 0.357 | 141 | Gleasonian |
| Stream_dia_Vantaa | 630 | -5.30 | 0.001 | 854 | 42 | 17591 | 1.89 | 0.058 | 11561 | 3185 | 2.76 | 0.001 | 115 | Q-Clementsian |
| Stream_bry_Kemij | 343 | -0.92 | 0.350 | 377 | 37 | 13193 | 1.18 | 0.230 | 9661 | 2971 | 2.13 | 0.001 | 29 | Random |
| Stream_bry_Muonio | 125 | -4.31 | 0.001 | 227 | 24 | 5071 | 0.87 | 0.380 | 4034 | 1185 | 1.85 | 0.008 | 34 | Q-Clementsian |
| Stream_bry_Utsj | 228 | -1.92 | 0.054 | 272 | 23 | 8128 | 2.39 | 0.016 | 4499 | 1517 | 3.11 | 0.001 | 41 | Random |

* An outlier (coherence Z and turnover Z scores) dataset that was not include in the comparative analysis.

Appendix S5. Variation among the main taxonomic groups in the Z-scores of coherence, the Z-scores of turnover, the index of boundary clumping, and the three components of multiple site beta diversity (i.e. Sorensen, Simpson and Nested). Abbreviations: alg = algae; bac = bacteria; inv = invertebrates; mp = macrophytes; ver = fish.

Appendix S6. Correlations between the Z-scores of coherence, the Z-scores of turnover, the index of boundary clumping, and the three components of multiple site beta diversity (i.e. Sorensen, Simpson and Nested). Pearson correlations coefficients (*r*) between the indices are shown in the upper diagonal.

Appendix S7. The 44 metacommunities plotted in the “metacommunity characteristics space” of Principal Coordinates Analysis (PCoA). PCoA was based on Gower distance matrix on the five ecological characteristics of the metacommunities (i.e. body size, trophic group, ecosystem type, life form and dispersal mode). Large black dot refers to a position of a metacommunity in the PCoA ordination space. Note that multiple metacommunities have the same score; hence, the boxes connect some metacommunities to the same score. Observed metacommunity types are shown by different colours.
